# Management of Cancer-Associated Thrombosis: Unmet Needs and Future Perspectives

**DOI:** 10.1055/s-0041-1736037

**Published:** 2021-08-31

**Authors:** Anna Falanga, Grégoire Le Gal, Marc Carrier, Hikmat Abdel-Razeq, Cihan Ay, Andrés J. Muñoz Martin, Ana Thereza Cavalcanti Rocha, Giancarlo Agnelli, Ismail Elalamy, Benjamin Brenner

**Affiliations:** 1 Department of Medicine and Surgery, School of Medicine, University of Milano-Bicocca, Monza, Italy; 2 Department of Immunohematology and Transfusion Medicine, Thrombosis and Hemostasis Center, Hospital Papa Giovanni XXIII, Bergamo, Italy; 3 Department of Medicine, Ottawa Hospital Research Institute, University of Ottawa, Ottawa, Ontario, Canada; 4 Department of Medicine, King Hussein Cancer Center, Amman, Jordan; 5 Clinical Division of Haematology and Haemostaseology, Department of Internal Medicine, Comprehensive Cancer Center Vienna, Medical University of Vienna, Vienna, Austria; 6 Department of Obstetrics and Gynecology, I. M. Sechenov First Moscow State Medical University, Moscow, Russia; 7 Medical Oncology Department, Hospital General Universitario Gregorio Marañón, Universidad Complutense, Madrid, Spain; 8 Departamento de Saúde da Família, Faculdade de Medicina da Bahia, Universidade Federal da Bahia – UFBA, Salvador, BA, Brazil; 9 Internal Vascular and Emergency Medicine – Stroke Unit, University of Perugia, Perugia, Italy; 10 Hematology and Thrombosis Centre, Hôpital Tenon, INSERM U938, Sorbonne Université, AP-HP, Paris, France; 11 Department of Hematology, Rambam Health Care Campus, Haifa, Israel

**Keywords:** cancer, thrombosis, DVT, VTE, PE, thrombocytopenia, LMWH, DOACs

## Abstract

Patients with cancer are at a high risk of symptomatic venous thromboembolism (VTE), which is a common cause of morbidity and mortality in this patient population. Increased risk of recurrent VTE and bleeding complications are two major challenges associated with therapeutic anticoagulation in these patients. Long-term therapy with low-molecular-weight heparins (LMWHs) has been the standard of care for the treatment of cancer-associated VTE given its favorable risk–benefit ratio in comparison with vitamin K antagonists. Direct oral anticoagulants (DOACs), which offer the convenience of oral administration and have a rapid onset of action, have recently emerged as a new treatment option for patients with cancer-associated thrombosis (CT). Randomized clinical trial data with head-to-head comparisons between DOACs and LMWHs showed that overall, DOACs have a similar efficacy profile but a higher risk of bleeding was observed in some of these studies. This review aims to identify unmet needs in the treatment of CT. We discuss important considerations for clinicians tailoring anticoagulation (1) drug–drug interactions, (2) risk of bleeding (e.g., gastrointestinal bleeding), (3) thrombocytopenia, hematological malignancies, (4) metastatic or primary brain tumors, and (5) renal impairment. Additional research is warranted in several clinical scenarios to help clinicians on the best therapeutic approach.

## Introduction

Thromboembolism is a common complication associated with malignant diseases and is the second leading cause of mortality in patients with cancer. 1 2 Between 1997 and 2017, the risk of venous thromboembolism (VTE) in cancer patients increased threefold overall and sixfold in cancer patients treated with chemotherapy or targeted therapy. 3 When compared with the general population for a period of over 12 months, cancer patients had a ninefold higher cumulative incidence of VTE. 3 Furthermore, those who develop VTE at diagnosis of cancer or within a year of diagnosis have worse disease prognosis compared with cancer patients without VTE. 4 Common thrombotic complications in cancer patients include arterial thromboembolism (ATE) or VTE and disseminated intravascular coagulation. 5 The pathogenesis of cancer-associated thrombosis (CT) is complex and involves abnormalities in each component of Virchow's triad: stasis of blood flow, endothelial injury, and hypercoagulability. Treatments of cancer with radiotherapy, systemic chemotherapy, newer molecular targeted drugs, and immune checkpoint inhibitor therapies as well as the use of central venous catheters also lead to an increased risk of VTE and ATE. 6 7 8 9

Different cancer types carry different levels of VTE risk. For instance, hematological malignancies, lung, pancreas, stomach, colorectal, and brain cancers are associated with a high risk of thrombosis, while prostate and breast cancers are associated with a lower risk of clot formation. 10 11 12 An increased risk of recurrent VTE and bleeding complications among cancer patients complicates the management of VTE compared with patients without cancer. 13 Hence, in cancer patients with VTE, a better understanding and balancing of the associated risks and benefits are required to individualize a therapeutic anticoagulation strategy that can lead to a significant improvement in clinical outcomes. Low-molecular-weight heparins (LMWHs) and direct oral anticoagulants (DOACs) are currently recommended for the short- and long-term management of CT in clinical practice guidelines. 14 15 16 Recently, several trials examined the use of DOACs in the treatment of CT and support the use of DOACs as potential alternatives to LMWH. 17 However, oral anticoagulation treatment may be challenging in patients at risk of drug–drug interactions, severe bleeding, and thrombocytopenia. 18 19 20 21 22 In this review article, we present our current understanding of the treatment of established CT. Further, we attempt to highlight challenging clinical scenarios, including management of anticoagulation in patients with gastrointestinal (GI), hematological, and central nervous system (CNS) malignancies; thrombocytopenia; VTE recurrence during anticoagulation; drug–drug interactions; and renal impairment, which continue to remain as unmet medical needs in the treatment of CT.

## Challenges Associated with Treatment of Cancer-Associated Thrombosis

Two major challenges in the management of VTE in cancer patients are recurrent VTE and major bleeding.

Recurrent VTE despite anticoagulation is common among cancer patients. Reasons for recurrence may be due to patient- (noncompliance or poor injection technique), tumor- (vascular compression, high-risk tumors, and gastric or pancreatic adenocarcinoma), or treatment-related factors. 5 Recent data suggest that interruption of periprocedural anticoagulation leads to increased postoperative rates of VTE recurrence and major bleeding in patients with CT compared with the noncancer patients. 23 The management of recurrent VTE is controversial, but clinicians may consider an alternative anticoagulant regimen, an increased dose of LMWH, or adding a vena cava filter to LMWH. 16 24

Anticoagulation therapy in general increases the risk of bleeding in cancer patients compared with that in noncancer patients, particularly in patients with GI tract, genitourinary tract, and gynecologic malignancies. Given that recurrent VTE and major bleeding complications are associated with significant morbidity and a decrease in quality of life in patients with cancer, it is important to weigh the risks and benefits to minimize these complications while deciding on which anticoagulant should be used. 25 26

## Recommended Treatment for Cancer-Associated Thrombosis

As discussed above, treatment choices for the acute and extended management of CT must be tailored to not only prevent recurrent VTE but also avoid bleeding complications—the most severe adverse event of anticoagulation therapy.

### Low-Molecular-Weight Heparins

Until 2002, vitamin K antagonists (VKAs), such as warfarin, were primarily used for the management of VTE in patients with cancer. 27 Later on, results of the CLOT trial led to the use of LMWH as the first-line choice for the treatment of CT. 28 Dalteparin was shown to be more effective than VKA with less recurrent VTE occurring in patients receiving dalteparin (27/336 vs. 53/336; hazard ratio [HR]: 0.48, 95% confidence interval [CI]: 0.30–0.77; *p* = 0.002). The study also showed that safety outcomes were similar with LMWH compared with VKA (major bleeding occurred, 6 vs. 4%; *p* = 0.27), and no difference in overall survival was observed between groups (overall mortality, 39 vs. 41%; *p* = 0.53). 28 Another clinical trial that reproduced the results of CLOT was the CATCH (tinzaparin vs. VKA) trial. 29 In the CATCH trial, the primary outcome of recurrent VTE was 7.2% in the tinzaparin group versus 10.5% in the warfarin group (HR: 0.65, 95% CI: 0.41–1.03; *p* = 0.07); similarly, no major difference was observed between groups with respect to major bleeding (2.7 vs. 2.4%; HR: 0.89, 95% CI: 0.40–1.99; *p* = 0.77) and overall survival (death occurred in 33.4 vs. 30.6%; HR: 1.08, 95% CI: 0.85–1.36; *p* = 0.54). 29 Another LMWH, enoxaparin, was first evaluated in CANTHANOX, a randomized open-label trial. 30 CANTHANOX compared enoxaparin and VKA for the secondary prevention of VTE in patients with cancer. The primary composite outcome of major bleeding or recurrent VTE was observed in 21.1% (95% CI: 12.3–32.4) and 10.5% (95% CI: 4.3–20.3; *p* = 0.09) of patients receiving warfarin and enoxaparin, respectively. 30 ONCENOX was another randomized open-label study that compared the safety and efficacy of enoxaparin alone with that of initial enoxaparin followed by warfarin in secondary prevention of VTE in patients with CT. 31 The three-armed ONCENOX (enoxaparin 1 mg twice daily, enoxaparin 1.5 mg daily, and VKA) study demonstrated no major differences between enoxaparin and VKA groups in terms of recurrent VTE (3.4 vs. 3.1 vs. 6.7%) and major bleeding (6.5 vs. 11.1 vs. 2.9%). 31 Three meta-analyses that evaluated the use of LMWH in VTE with cancer further confirmed the benefits of LMWH in the management of CT. 32 33 34 International guidelines have recommended the use of LMWH for short- and long-term management of CT. 15 16 Recommended duration of anticoagulation should be at least 3 to 6 months, and the decision of treatment beyond the initial 6 months should be on case-by-case basis considering the risk of recurrent VTE and major bleeding. 15 16

### Direct Oral Anticoagulants

More recently, DOACs, including direct thrombin inhibitors (dabigatran) and direct factor Xa inhibitors (rivaroxaban, apixaban, and edoxaban), were shown to have a favorable efficacy and safety profile as compared with VKAs in studies with acute VTE. 35 Nevertheless, cancer patients were under-represented in these VKA-controlled clinical trials, given that LMWHs were the standard of care for CT at the time these studies were conducted. Given that DOACs do not require dose adjustment after laboratory monitoring and avoid the burden of daily LMWH injections, several trials have examined or are currently ongoing to assess their safety and efficacy in the treatment of CT. 18 19 20 21 22 36

### Clinical Trials Comparing Direct Oral Anticoagulants versus Low-Molecular-Weight Heparins

Across all randomized clinical trials (RCTs) that evaluated DOACs against LMWH, the selection of dalteparin as a comparator was appropriate as the drug is approved for the long-term treatment of patients with CT both in the United States and Europe. 19 20 21 22 37 38 In all studies, subcutaneous dalteparin was given at a dose of 200 IU/kg once daily for the first month, followed by a dose reduction to 150 IU/kg for months 2 through 6. 19 20 21 22 In 2018, results of two clinical trials, Hokusai VTE Cancer and SELECT-D, were published. 21 22 Hokusai VTE Cancer was a multinational, Prospective, Randomized, Open-label, Blinded endpoint Evaluation (PROBE), noninferiority trial, comparing edoxaban and dalteparin for the treatment of CT for a minimal duration of 6 and up to 12 months. 39 Edoxaban was noninferior to dalteparin with respect to the primary outcome of composite measure of recurrent VTE or major bleeding (12.8 vs. 13.5%; HR: 0.97 [95% CI: 0.70–1.36]; *p* = 0.006 for noninferiority and *p* = 0.87 for superiority). 21 Edoxaban was associated with a nonsignificant (7.9 vs. 11.3%; HR: 0.71 [95% CI: 0.48–1.06]; *p* = 0.09) lower risk of VTE recurrence but a higher risk of major bleeding (6.9 vs. 4%; [HR: 1.77; 95% CI: 1.03–3.04; *p* = 0.04]), mostly due to more upper GI bleeding events in patients with GI tumors. The incidence of clinically relevant non-major bleeding (CRNMB) was similar between groups. 21 SELECT-D, an open-label, randomized, multicenter pilot study, compared rivaroxaban (direct factor Xa inhibitor) with dalteparin for the treatment of CT. 22 The primary outcome of 6-month risk of recurrent VTE was 4% with rivaroxaban and 11% with dalteparin (HR: 0.43; 95% CI: 0.19–0.99). 22 The risk of major bleeding was not significantly different between groups (6.0 vs. 4.0%; HR: 1.83, 95% CI: 0.68–4.96). The risk of CRNMB was higher with rivaroxaban (13 vs. 4%). Patients with esophageal and gastroesophageal cancer were excluded during the trial due to apparent imbalance in major bleeding rates (rivaroxaban vs. dalteparin, 36 vs. 11%). 22 A year later, the ADAM VTE trial compared apixaban with dalteparin for the treatment of CT. 20 In this randomized, open-label, investigator-initiated trial, major bleeding was the primary outcome and occurred in 0% of patients in the apixaban treatment arm compared with 1.4% of patients in the dalteparin group. 20 The rate of the secondary outcome, recurrent VTE, was significantly lower in the apixaban group compared with that in the dalteparin group (0.7 vs. 6.3%, HR: 0.099; 95% CI: 0.013–0.780; *p* = 0.0281). Incidence of CRNMB was similar in both groups (6%). 20 Another randomized controlled trial (CARAVAGGIO) evaluating apixaban for CT treatment was recently published. CARAVAGGIO was a large investigator-initiated, multinational, prospective, randomized PROBE, noninferiority clinical trial comparing 6-month treatment with oral apixaban and subcutaneous dalteparin in cancer patients with VTE. 18 19 The primary outcome of objectively confirmed recurrent VTE occurred in 5.6% of patients in the apixaban group compared with 7.9% in the dalteparin group (HR: 0.63; 95% CI: 0.37–1.07; *p* < 0.001 for noninferiority and *p* = 0.09 for superiority). 19 The incidence of major bleeding was similar in both treatment groups (3.8 vs. 4%; HR: 0.82; 95% CI: 0.40–1.69; *p* = 0.60). Rate of major GI bleeding was 1.9 and 1.7% in the apixaban and dalteparin groups, respectively; major non-GI bleeding occurred in 11 (1.9%) patients in the apixaban group and in 13 (2.2%) patients in the dalteparin group. There was no significant difference observed in the incidence of CRNMB between groups (9.0 vs. 6.0%; HR: 1.42; 95% CI, 0.88–2.30). 19 Results from four completed clinical trials (Hokusai VTE Cancer, SELECT-D, ADAM VTE, and CARAVAGGIO) were pooled in a recently published meta-analysis to update the evidence for treatment of CT. 17 40

**Fig. 1:**
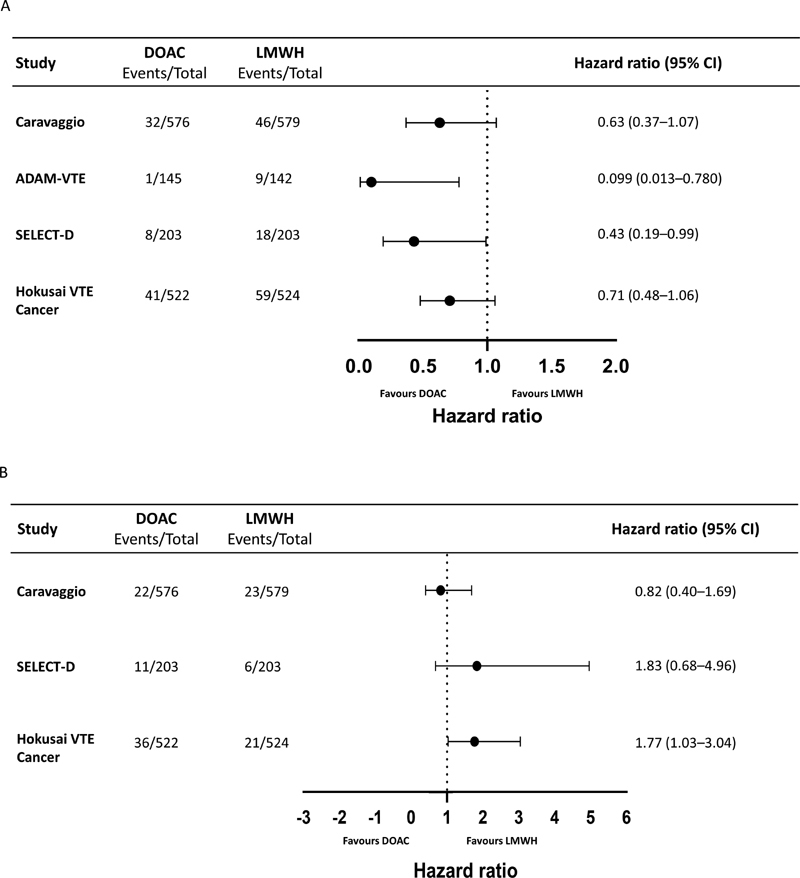
( **A** ) Forest plot of hazard ratios for recurrent VTE in clinical trials evaluating DOAC vs. LMWH and ( **B** ) forest plot of hazard ratios for major bleeding in clinical trials evaluating DOAC vs. LMWH. CI, confidence interval; DOAC, direct oral anticoagulant, HR, hazard ratio; LMWH, low-molecular-weight heparin; VTE, venous thromboembolism

There is heterogeneity with respect to selection of patients among the four clinical trials. They differ in their primary outcomes, design, and type and stage of cancer included. These key differences and efficacy and safety results are highlighted in Table 1 . In summary, the results of the Hokusai VTE Cancer and SELECT-D trials revealed that DOACs tend to reduce the risk of recurrent VTE but increase the risk of major and CRNMB events, especially in patients with GI cancers. 17 In the ADAM VTE trial, the low rates of major bleeding could possibly be due to fewer participants with upper GI malignancy compared with the Hokusai VTE Cancer trial. 20 In the CARAVAGGIO study, apixaban was noninferior to dalteparin for VTE events without an increased risk of major bleeding ( Fig. 1 ). However, it is important to note that patients with other common high bleeding risk factors, such as brain metastases, leukemia, liver disease, thrombocytopenia (platelet count <75 × 10 ^9^ /L), creatinine clearance <30 mL/min, and concomitant antiplatelet therapy (up to 165 mg/day of aspirin was allowed), were excluded from the CARAVAGGIO study. 18 In addition, only 4% of patients with upper GI cancer who were at a high risk of bleeding were included in the CARAVAGGIO study. 18

**Table 1:** Data from randomized clinical trials comparing DOAC with LMWH

	Hokusai VTE Cancer ( *N* = 1,046) a		SELECT-D ( *N* = 406) a		ADAM-VTE ( *N* = 287) b		CARAVAGGIO ( *N* = 1,155) a	
Treatment	Edoxaban ( *n* = 522)	Dalteparin ( *n* = 524)	Rivaroxaban ( *n* = 203)	Dalteparin ( *n* = 203)	Apixaban ( *n* = 145)	Dalteparin ( *n* = 142)	Apixaban ( *n* = 576)	Dalteparin ( *n* = 579)
Study characteristics								
First author and year of publication	Raskob, 2018 21		Young, 2018 22		McBane, 2020 20		Agnelli, 2020 19	
Study design	Randomized open-label, noninferiority trial with blinded adjudication of outcomes		Randomized, open-label, multicenter pilot trial with blinded adjudication of outcomes		Randomized, open-label, investigator-initiated, multicenter, superiority trial		Randomized, controlled, investigator-initiated, open-label, noninferiority trial with blinded adjudication of outcomes	
Primary outcome	Composite of recurrent VTE or major bleeding at 12 months		Recurrent VTE at 6 months		Major bleeding at 6 months		Objectively confirmed recurrent VTE at 6 months	
Treatment duration	12 mo		6 mo		6 mo		6 mo	
Type of VTE	Symptomatic or incidental VTE		Symptomatic or incidental VTE		Symptomatic or incidental VTE		Symptomatic or incidental VTE	
Type of cancer excluded	Basal cell/squamous cell cancer of the skin		Basal cell/squamous cell cancer of the skin		Basal cell/squamous cell cancer of the skin		Basal cell/squamous cell cancer of the skin, brain metastases/primary tumors, acute leukemia	
ECOG performance status excluded	ECOG: 3–4		ECOG: 3–4		ECOG: 3–4		ECOG: 3–4	
Patient characteristics								
Age (y)	64.3 (11.0)	63.7 (11.7)	Median: 67	Median: 67	64.4 (11.3)	64.0 (10.8)	67.2 (11.3)	67.2 (10.9)
Metastatic cancer, *n* (%)	554 (53.0)		236 (58.1)		193 (64.3)		785 (68.0)	
GI cancer, *n* (%)	305 (29.2)		177 (43.6)		105 (35.0)		375 (32.5)	
Hematological cancer, *n* (%)	111 (10.6)		31 (7.6%)		28 (9.3)		85 (7.4)	
Brain tumor	–		3 (0.7)		8 (2.7)		0 (0.0)	
Efficacy and safety outcomes								
Recurrent VTE (%)	7.9	11.3	4.0	11.0	0.7	6.3	5.6	7.9
HR, (95% CI)	0.71 (0.48–1.06)		0.43 (0.19–0.99)		0.099 (0.013–0.780)		0.63 (0.37–1.07)	
Major bleeding (%)	6.9	4.0	6.0	4.0	0.0	1.4	3.8	4.0
HR, (95% CI)	1.77 (1.03–3.04)		1.83 (0.68–4.96)		(Not estimable)		0.82 (0.40–1.69)	
Major GI bleeding (%)	3.8	1.1	3.4	2.0	–	–	1.9	1.7
Major GU bleeding (%)	1.0	0	0.5	0	–	–	0.7	0.2
CRNMB (%)	14.6	11.1	12.3	3.4	6.2	4.2	9.0	6.0
HR, (95% CI)	1.38 (0.98–1.94)		3.76 (1.63–8.69)		0.931 (0.43–2.02)		1.42 (0.88–2.30)	
Intracranial bleeding (%)	0.4	0.8	–	–	0	0.7	0.0	0.3

Abbreviations: CI, confidence interval; CRNMB, clinically relevant nonmajor bleeding; DOAC, direct oral anticoagulant; ECOG, Eastern Cooperative Oncology Group; GI, gastrointestinal; GU, genitourinary; HR, hazard ratio; LMWH, low-molecular-weight heparin; *N* , number of total subjects in a trial; *n* , number of total subjects in a group; VTE, venous thromboembolism.

a Intention-to-treat population for Hokusai VTE Cancer, SELECT D, CARAVAGIO.

b Per protocol population for ADAM VTE.

These clinical trial results concluded that edoxaban, rivaroxaban, and apixaban were noninferior to LMWH in preventing VTE recurrence and revealed that DOACs may be an appropriate alternative to LMWH for the treatment of CT. 41 Based upon the results of the Hokusai VTE Cancer and SELECT-D studies, DOACs (edoxaban and rivaroxaban) were incorporated in the updated clinical practice guidelines of the International Initiative on Thrombosis and Cancer and the American Society of Clinical Oncology. *Guidance statement: For patients who do not have a high risk of gastrointestinal or genitourinary bleeding, a regimen of rivaroxaban (in the first 10 days) or edoxaban (started after at least 5 days of parenteral anticoagulation) can also be used for the initial treatment of established VTE in patients with cancer when creatinine clearance is ≥30 mL/min* . 15 16

Amidst potential safety concerns reported in some of the previously published RCTs, the similar risks of bleeding complications between dalteparin and apixaban reported in the CARAVAGGIO trial are of interest. 19 With this attractive result of similar safety with respect to the incidence of major bleeding and CRNMB, physicians may consider apixaban as the safest DOAC in patients with GI malignancies. However, as there was heterogeneity between the trials and there is no direct comparison between different DOACs, it is inappropriate to conclude that one DOAC is better than another. Additional studies in patients at a high risk of bleeding (upper GI cancers and genitourinary tumors) are needed before apixaban can be recommended over other DOACs. 19

### Treatment of Incidental Venous Thromboembolism

Incidental VTE is defined as deep vein thrombosis (DVT) or pulmonary embolism (PE) discovered on diagnostic imaging performed for a reason other than clinical suspicion of VTE. Patients with incidental VTE have a similar risk of recurrent VTE despite anticoagulation compared with patients with symptomatic events. 42 43 Nevertheless, to date, there is no specific risk assessment model to predict incidental VTE, and many vulnerable patients likely remain undiagnosed until the occurrence of symptoms, which could have been otherwise avoided with timely initiation of anticoagulation therapy. Clinical practice guidelines suggest that patients with incidentally diagnosed DVT or PE should be treated similarly to those diagnosed with VTE based on symptoms. Isolated subsegmental PE and visceral vein thrombi are exceptions where decisions can be made on a case-by-case basis. A recent meta-analysis focused on incidental VTE from the four RCTs that evaluated DOACs against LMWH for the treatment of CT. Incidental VTE was observed in 30.0% of patients, ranging from 19.9% in the CARAVAGGIO trial 19 and 27.8% in the SELECT-D trial 22 to 32.5% in the Hokusai VTE Cancer trial, 21 with no reported events in the ADAM VTE trial. 20 The risk of recurrent VTE was similarly reduced with DOACs in patients with incidental and symptomatic VTE (incidental: relative risk [RR], 0.58 [95% CI: 0.28–1.18]; *p* = 0.134; *I* ^2^ = 0.0%; symptomatic: RR, 0.76 [95% CI: 0.55–1.07]; *p* = 0.118; *I* ^2^ = 0.0%), whereas the risk of major bleeding was similar between patients with incidental and symptomatic VTE (incidental: RR, 1.11 [95% CI: 0.53–2.32]; *p* = 0.785; *I* ^2^ = 0.0%; symptomatic: RR 1.50 [95% CI: 0.55–4.07]; *p* = 0.422; *I* ^2^ = 67.4%). 17 This observation supports the recommendation of the same therapeutic approach for both symptomatic and incidental VTE.

### Duration of Anticoagulation and Treatment beyond 6 Months

Duration of anticoagulation in patients with CT is still a matter of debate. In the cancer setting, in patients who developed CT and had adequately been treated for 6 months, should anticoagulation be continued after 6 months? Or is it safe to discontinue anticoagulants? These are some of the unanswered questions related to the treatment of CT beyond 6 months. Given the increased risk of VTE recurrence, continuing anticoagulation beyond 6 months should be considered for selected patients. Guidelines recommend that for patients with ongoing metastatic disease and on continued chemotherapy, treatment should be continued with LMWH, DOACs, or VKAs beyond 6-month anticoagulation. Termination or continuation of anticoagulation may be based upon the individual evaluation of the benefit–risk ratio, tolerability, drug availability, patient preference, and cancer activity. 15 The DALTECAN and TiCAT studies evaluated the safety of dalteparin and tinzaparin beyond 6 months in patients with CT. 44 45 In the DALTECAN study, the incidence of major bleeding was 0.7% (95% CI: 0.3–1.4) during the 7- to 12-month study period compared with 1.7% (95% CI: 1.1–2.4) during the initial 6 months of the study. 44 Similar results were observed in the TiCAT study; clinically relevant bleeding during months 1 to 6 and 7 to 12 was 0.9% (95% CI: 0.5–1.6) and 0.6% (95% CI: 0.2–1.4) per patient and month, respectively. 45 The above-mentioned results support the safe use of LMWH in patients with CT beyond 6 months. Recent RCTs that evaluated DOACs versus LMWH for CT treatment, including the CARAVAGGIO trial, reported only 6-month rates of outcome variables. 19 20 21 22 In the Hokusai VTE Cancer trial, enrolled patients were followed up for up to 12 months and demonstrated that no significant differences were observed in the rate of the primary composite endpoint of recurrent VTE and/or major bleeding between 6 and 12 months (edoxaban vs. dalteparin: 2.4 vs. 2.2%; unadjusted HR: 1.05; 95% CI: 0.36–0.35). 46 In the SELECT-D trial, patients with PE or residual DVT at 6 months after randomization were considered for the second randomization to evaluate efficacy and safety of rivaroxaban at 12 months. 47 The Kaplan–Meier estimates of VTE recurrence were lower in the rivaroxaban arm (4%) compared with the placebo arm (14%) at month 12; however, the difference was not statistically significant due to the small sample size and consequently, the lack of statistical power. Two (5%) patients experienced major bleeding in the rivaroxaban arm compared with none in the placebo arm. The rate of CRNMB was 4% in the rivaroxaban arm and 0% in the placebo arm. 47 Robust clinical trial data with additional studies are warranted to evaluate the clinical benefit of DOACs for extended treatment duration. The APIxaban Cancer-Associated Thrombosis (API-CT; NCT03692065) 48 trial is ongoing and will provide more insight into these important clinical questions related to extended anticoagulation with DOACs. API-CT is a randomized, double-blind, multicenter, international, prospective, and parallel-group study to compare the two dose regimens of apixaban (2.5 vs. 5 mg) for the prevention of recurrent VTE in patients with cancer who have completed at least 6 months of anticoagulant treatment. 48

## Factors to Consider While Tailoring VTE Management in Special Population

### Inferior Vena Cava Filters

International guidelines suggest that in patients with acute VTE, inferior vena cava (IVC) filters may be considered when anticoagulant treatment is contraindicated (such as recent major surgery, tumors at high risk of hemorrhage, perioperative period, and thrombocytopenia with a very low platelet count) or when recurrence occurs despite optimal anticoagulation. 49 For patients with acute VTE who are actively bleeding or have severe, prolonged thrombocytopenia for which anticoagulation with platelet transfusion cannot be achieved, retrievable IVC filters may be considered on a case-by-case basis. 50 However, the risk of recurrent VTE is generally high in patients having IVC filters inserted and with no evidence of improvement in survival. Removal of the IVC filter is strongly recommended when the patient is back on anticoagulation and no longer bleeding or at high risk of bleeding complications.

### Recurrent Venous Thromboembolism during Anticoagulation

Despite anticoagulation, approximately 20% of patients with cancer exhibit recurrent VTE. 51 As per the RIETE registry (Registro Informatizado de Pacientes con Enfermedad TromboEmbólica [Computerized Registry of Patients with Venous Thromboembolism]) (a large database with approximately 99,000 patients with VTE), 52 the risk factors associated with recurrent VTE include younger age (<65 years), newly diagnosed cancer (<3 months), site of cancer (ovarian cancer, stage IV pancreatic cancer, brain tumor, lung cancer, and myeloproliferative neoplasms), stage of cancer (advanced/metastatic), and histology of cancer (adenocarcinoma). 53 54 55

The ability to assess which patients are at a higher risk of recurrent VTE despite anticoagulation would be useful to select the most appropriate treatment while balancing the risk of bleeding. Louzada et al developed the “Ottawa risk stratification model” for evaluation of the risk of VTE recurrence in patients with cancer. 56 When the risk score was applied and validated with the clinical trial data from the CLOT and CANTHANOX trials, it showed that a patient with a score of <0 had a low risk (5.1%), patients with a score of “0” had an intermediate risk (9.8%), and those with a score of ≥1 had a high risk (15.8%) of recurrent VTE. 56 However, in another prospective cohort study, the Ottawa score failed to predict the risk of VTE recurrence despite curative anticoagulation with LMWH in patients with CT. 57 More studies are required to validate the Ottawa score and to determine the best treatment option for cancer patients at risk of recurrent VTE.

A systematic review and meta-analysis of clinical trials evaluating DOACs versus LMWH for the treatment of CT showed that the risk of recurrent VTE was nonsignificantly lower with DOACs (5.6%) compared with LMWH (8.3%). 58 Currently, there are minimal data available on the management of recurrent VTE in patients with CT. The treatment strategies suggested by the guidelines are based solely upon retrospective studies and expert opinions. 59 60 61 62

### Thrombocytopenia

Comorbid conditions such as thrombocytopenia (defined as platelet count <100 × 10 ^9^ /L) is a common complication affecting patients on anticancer therapies. This increases the risk of bleeding particularly in patients with hematological malignancies and further complicates the treatment of CT. Despite the increased risk of bleeding, the risk of VTE is not reduced in these patients. The Scientific and Standardization Committee of the International Society on Thrombosis and Haemostasis recommends that full doses of anticoagulant can be used for the treatment of established VTE if the platelet count is >50 × 10 ^9^ /L and there is no evidence of bleeding. 15 50 In patients with severe thrombocytopenia (<50 × 10 ^9^ /L), two management strategies are proposed. For patients with acute VTE (<30 days since the diagnosis of the event), full-dose anticoagulation with transfusion support to maintain platelet levels to 40 to 50 × 10 ^9^ /L is suggested. For patients with lower risk events (distal DVT, incidental subsegmental PE, catheter-related thrombosis, and other low-risk features), dose-modified anticoagulation with a half-therapeutic or the prophylactic dose of LMWH is suggested. Anticoagulation should be withheld if the platelet count is <25 × 10 ^9^ /L. For patients with subacute VTE (beyond 30-day period since the diagnosis), decreased dosing (50% or prophylactic LMWH) is recommended for a platelet count of 25 to 50 × 10 ^9^ /L and temporary discontinuation for <25 × 10 ^9^ /L. LMWH is currently the preferred drug of choice in these patients. 50 Data on the use of DOACs in patients with a platelet count of <50 × 10 ^9^ /L is scarce. 19 20 21 22 In the Hokusai VTE Cancer trial in which participants were randomized to edoxaban or dalteparin, patients were excluded if they had a platelet count of <50,000/mL. 21 The platelet count cutoff of <50 × 10 ^9^ /L was used to withhold anticoagulation with rivaroxaban in the SELECT-D and with apixaban in the CARAVAGGIO trial. 19 22

### Drug–Drug Interactions

Drug–drug interaction with chemotherapeutic agents, hormonal therapy, and immune-modulating agents is a common concern with oral anticoagulants, including both warfarin and DOACs. 63 DOACs are substrates of CYP3A4 and P-gp. Interaction of other therapies that influence the activities of CYP3A4 and/or P-gp could lead to altered metabolism and/or elimination, eventually impacting the plasma concentrations of DOACs. 64 65 This was supported by data from a recent observational study where bleeding risk was increased when DOACs were given in combination with P-gp/moderate CYP3A4 inhibitors (amiodarone, dronedarone, diltiazem, and verapamil) compared with DOACs use alone. Another study that examined concomitant use of DOACs with clarithromycin or azithromycin revealed that the use of clarithromycin, which is a potent inhibitor of CYP3A4 and P-gp, led to an increased rate of hemorrhage requiring hospitalization compared with azithromycin, a mild inhibitor of CYP3A4 and P-gp. 66 67 Antineoplastic agents that might influence the efficacy and safety of DOACs are paclitaxel, bicalutamide, enzalutamide, certain tyrosine kinase inhibitors, and abiraterone, and supportive care medications include dexamethasone, prednisone, azole antifungals, and neurokinin-1 antagonists. 68 While a recent posthoc analysis on participants in the CARAVAGGIO study showed comparative efficacy and safety of apixaban in patients treated or not treated with anticancer agents. However, the study had several limitations, and further data and evidence on the concomitant use of DOACs and anticancer agents are needed. 69 Potential drug–drug interaction should be checked prior to using a DOAC, and its usage should be carefully considered if potential drug–drug interactions are anticipated; however, clinical implications of such interactions are widely unknown for most of the drugs.

### Gastrointestinal Malignancies

Among the different subtypes of malignancy, GI cancers have an incidence of more than 5% of clinically relevant VTE (pancreatic [16–22%], gastric [12–17%], and colorectal [8–12%] cancers). 70 71 72 Most clinical trials assessing efficacy and safety of anticoagulant therapies in cancer patients did not differentiate between different cancers, resulting in limited data for specific tumor types, including GI cancers. 73 Two of the above-discussed RCTs (Hokusai VTE Cancer and SELECT-D) showed that the risk of major bleeding seems to be higher in the subgroup of patients with GI cancers treated with DOACs compared with LMWH. 19 20 21 22 A recent meta-analysis of the four RCTs mentioned above also included a subgroup analysis of the Hokusai VTE Cancer and SELECT-D trials in patients with GI cancer, including colorectal, gastric, gastroesophageal, pancreatic and hepatobiliary cancer ( *N* = 1,452). 17 The risk of major bleeding at 6 months was significantly increased in patients with GI cancer treated with DOACs compared with LMWH (9.3 vs. 4.0%; [RR]: 2.30 [95% CI: 1.08–4.88]; *p* = 0.031). In contrary, the difference in the incidence of major bleeding between treatment groups was small in patients with non-GI malignancies (3.4 vs. 2.9%; RR: 1.22 [95% CI: 0.60–2.48]; *p* = 0.580). 17 These findings suggest that clinicians should be careful in using DOACs for patients with active or nonsurgically treated GI tumors.

### Central Nervous System Malignancies

Within the cancer population, patients with CNS malignancies have a particularly high incidence of VTE (20–30%). 74 75 Early diagnosis and effective anticoagulation in patients with primary brain tumors and brain metastases are serious concerns. Management of VTE in these patients is complicated by multiple factors, such as compliance, drug interactions, and, most importantly, the chances of developing intracranial hemorrhage (ICH). Data on the safety of anticoagulation in these patients are scarce. Inclusion of patients with brain tumors in recent RCTs comparing DOACs versus LMWH was very limited. In the Hokusai VTE Cancer trial, 6.5% (2/31) and 9.3% (4/43) of patients with brain tumors on edoxaban and dalteparin, respectively, were diagnosed with a major hemorrhage event. 21 76 In the CARAVAGGIO study with apixaban, patients with brain tumors were excluded. 19 Observational data suggest that intracranial bleeding is more common in patients with primary CNS tumors than in patients with intracranial metastases. Guidelines recommend to use LMWH or DOACs for the treatment of established CT in patients with brain tumors. 15 16 A retrospective comparative cohort study including patients with either primary brain tumors ( *N* = 67) or secondary brain metastases ( *N* = 105) did not report any increase in the incidence of ICH with the use of DOACs ( *N* = 42) compared with LMWH ( *N* = 131). 77 In the primary brain tumor cohort, the cumulative incidence of any ICH was 0% versus 36.8% (95% CI: 22.3–51.3%) in DOACs versus LMWH, respectively. In the brain metastasis cohort, DOACs were not associated with a higher risk of any ICH relative to enoxaparin, 27.8% (95% CI: 5.5–56.7%) compared with 52.9% (95% CI: 37.4–66.2%). 77 However, the authors reported that selection bias could be a potential reason for the high risk of hemorrhage in patients treated with LMWH. 77 A lately published study suggested that in patients with brain metastases, the safety of DOACs and LMWH was similar. 78 More clinical trial data are warranted in these patients to determine the appropriate treatment option.

### Hematological Malignancies

The pharmacologic treatment of VTE in patients with hematological malignancies is challenging due to severe thrombocytopenia that can complicate the course of treatment. Because patients with hematological malignancies are included at a very low rate in clinical trials evaluating CT treatment, including in RCTs evaluating DOACs versus LMWH, there are not enough data or guidance related to the management of VTE in this population. In the CARAVAGGIO study, patients with leukemia were excluded. 19 Therefore, hematologists refer to the guidelines produced for patients with solid cancers. 15 79 Efficacy of DOACs in patients with hematological cancers needs to be established.

### Renal Impairment

The risk of bleeding is high in cancer patients with renal impairment. Administration of LMWH at therapeutic doses in patients with creatinine clearance <30 mL/min may lead to drug accumulation and increased risk of bleeding. In the presence of severe renal failure (creatinine clearance <30 mL/min), guidelines recommend using unfractionated heparin followed by early VKA or LMWH adjusted to anti-Xa level for the treatment of established VTE. When LMWH is required to be used in patients with severe renal impairment, it is recommended to measure anti-Xa levels. 15 16 80 No clinical evidence or real-world data related to the safety of DOACs are available for the treatment of CT in patients with severe renal impairment. 15 16

## Conclusion

Management of thrombosis and bleeding in cancer patients is a multilayered issue and requires careful consideration of associated risks in each patient and robust clinical judgment. Despite a large amount of data and the understanding of the risks and benefits of the management of CT, there are still many unanswered questions in this unique and critically challenging patient population. Based upon the data available from recent studies, DOACs may be considered a treatment option for patients with CT, particularly those with (1) no increased risk of any major bleeding (GI bleeding especially); (2) no severe thrombocytopenia (platelets count <50 × 10 ^9^ /L); (3) not taking concomitant medications that can potentially trigger drug–drug interactions; (4) no recent brain, spinal, or ophthalmic surgery; and (5) without any comorbid conditions such as severe liver or renal disease.

In conclusion, DOACs represent an additional treatment option to LMWHs for patients with CT. Treatment should be individualized considering the risk of bleeding, drug–drug interactions, toxicity, and, above all, patient preferences and values. Future studies should take into consideration relevant factors, such as cancer type and activity, and are warranted in patients with intracranial, GI, and hematological malignancies.
