# Corrigendum: Management of Cancer-Associated Thrombosis: Unmet Needs and Future Perspectives

**DOI:** 10.1055/s-0045-1810091

**Published:** 2025-07-10

**Authors:** Anna Falanga, Grégoire Le Gal, Marc Carrier, Hikmat Abdel-Razeq, Cihan Ay, Andrés J. Muñoz Martin, Ana Thereza Cavalcanti Rocha, Giancarlo Agnelli, Ismail Elalamy, Benjamin Brenner

**Affiliations:** 1 Department of Medicine and Surgery, School of Medicine, University of Milano-Bicocca, Monza, Italy; 2 Department of Immunohematology and Transfusion Medicine, Thrombosis and Hemostasis Center, Hospital Papa Giovanni XXIII, Bergamo, Italy; 3 Department of Medicine, Ottawa Hospital Research Institute, University of Ottawa, Ottawa, Ontario, Canada; 4 Department of Medicine, King Hussein Cancer Center, Amman, Jordan; 5 Clinical Division of Haematology and Haemostaseology, Department of Internal Medicine, Comprehensive Cancer Center Vienna, Medical University of Vienna, Vienna, Austria; 6 Department of Obstetrics and Gynecology, I. M. Sechenov First Moscow State Medical University, Moscow, Russia; 7 Medical Oncology Department, Hospital General Universitario Gregorio Marañón, Universidad Complutense, Madrid, Spain; 8 Departamento de Saúde da Família, Faculdade de Medicina da Bahia, Universidade Federal da Bahia – UFBA, Salvador, BA, Brazil; 9 Internal Vascular and Emergency Medicine – Stroke Unit, University of Perugia, Perugia, Italy; 10 Hematology and Thrombosis Centre, Hôpital Tenon, INSERM U938, Sorbonne Université, AP-HP, Paris, France; 11 Department of Hematology, Rambam Health Care Campus, Haifa, Israel

## Corrigendum

It has been brought to the publisher's attention that the name of Grégoire Le Gal was incorrectly tagged in the metadata of the above article published in *TH Open* , Volume 5, Issue 3, e376–e386, on August 31, 2021 (doi: 10.1055/s-0041-1736037 ). The given name and surname tagging has now been corrected in the article metadata.

